# The impact of attention-deficit/hyperactivity disorder and specific learning disorders on academic performance in Spanish children from a low-middle- and a high-income population

**DOI:** 10.3389/fpsyt.2023.1136994

**Published:** 2023-04-12

**Authors:** Gemma Español-Martín, Mireia Pagerols, Raquel Prat, Cristina Rivas, Josep Antoni Ramos-Quiroga, Miquel Casas, Rosa Bosch

**Affiliations:** ^1^Servei de Psiquiatria, Vall d’Hebron Hospital Universitari, Barcelona, Spain; ^2^Grup de Psiquiatria, Salut Mental i Addiccions, Vall d’Hebron Institut de Recerca (VHIR), Vall d’Hebron Hospital Universitari, Barcelona, Spain; ^3^Departament de Psiquiatria i Medicina Legal, Universitat Autònoma de Barcelona, Bellaterra, Spain; ^4^Centro de Investigación Biomédica en Red de Salud Mental (CIBERSAM), Instituto de Salud Carlos III, Madrid, Spain; ^5^Programa MIND Escoles, Hospital Sant Joan de Déu, Institut de Recerca Sant Joan de Déu, Esplugues de Llobregat, Spain; ^6^Unitat de Farmacologia, Departament de Fonaments Clínics, Facultat de Medicina i Ciències de la Salut, Universitat de Barcelona (UB), Barcelona, Spain; ^7^Centre for Health and Social Care Research (CEES), University of Vic-Central University of Catalonia (UVic-UCC), Vic, Spain

**Keywords:** Strengths and Difficulties Questionnaire (SDQ), attention-deficit/hyperactivity disorder (ADHD), specific learning disorders (SLD), socioeconomic status (SES), academic performance

## Abstract

**Introduction:**

Past research has demonstrated that attention-deficit/hyperactivity disorder (ADHD), specific learning disorders (SLD), and socioeconomic status (SES) affect a host of educational outcomes. However, there are no studies examining whether SES moderates the association between these neurodevelopmental disorders (ND) and the academic achievement of children and adolescents. The present investigation examined the impact of ADHD and SLD on academic performance in 1,287 Spanish students aged 5–17 from a low-middle (LM)- and a high-income population, when adjusted for comorbidity and demographic factors that may influence educational functioning.

**Methods:**

Parents completed a questionnaire regarding demographic data along with the Strengths and Difficulties Questionnaire. Additionally, teachers provided information on learning difficulties trough the Protocol for Detection and Management of Dyslexia. Teacher’s Version. Academic performance across multiple domains (i.e., first language, foreign language, mathematics) was obtained from school records. ND were determined using standardized diagnostic methods based on the Diagnostic and Statistical Manual of Mental Disorders criteria. To examine the effects of ADHD and SLD on academic achievement and the potential moderating role of SES, a series of ordinal logistic regressions were conducted.

**Results:**

Emotional/behavioral problems, learning difficulties, and ND were more frequent among individuals from the LM-income population. After controlling for gender, age, parental divorce/separation, grade retention, frequency of screen use, and daily meals, both ADHD and SLD were associated with worse educational outcomes. Lower SES also increased the risk for academic impairment, although the interactions with ADHD or SLD were not significant.

**Conclusion:**

These findings indicate that ADHD and SLD exert a pervasive impact on academic performance across different socioeconomic backgrounds. Therefore, early detection and effective intervention strategies aimed at students with these ND are crucial to improve their educational functioning and mitigate the negative consequences related to academic problems.

## Introduction

Neurodevelopmental disorders (ND) emerge at an early age and affect normal development, producing delays in the expected social, emotional, language, and cognitive performance. Attention-deficit/hyperactivity disorder (ADHD) and specific learning disorders (SLD) are among the most common ND in school-age children worldwide, with prevalence estimates ranging from 2 to 7% and 5 to 15%, respectively (1–3). ADHD is characterized by high levels of inattention, hyperactivity, impulsivity, and disruptive behavior. Depending on the predominance of the symptoms, it can be classified into three clinical presentations: predominantly inattentive, predominantly hyperactive–impulsive, and combined if six or more symptoms of each type are present for at least 6 months. Children with SLD, on the other hand, exhibit significant and persistent difficulties in learning academic skills, such as reading, written expression, and/or mathematics, despite adequate instruction and intelligence. In particular, dyslexia, which is one of the most common SLD, involve deficits in word reading accuracy, reading fluency, and reading comprehension. Dysgraphia can manifest as impairment in spelling accuracy, grammar/punctuation, and clarity or organization of written expression. Finally, children with impairment in mathematics may show problems in basic number processing, arithmetic facts, calculation skills, and math reasoning (1).

Past research has demonstrated a strong negative relation between ADHD symptoms and academic outcomes in clinical and community samples, with attention problems being the primary predictor of poorer achievement from early childhood into adulthood (4–6). These associations remain even after controlling for intelligence, psychiatric comorbidity, and socioeconomic status (SES) (7–9). Specifically, children with ADHD perform worse on standardized academic tests, have lower grade point average (GPA), and increased rates of absenteeism, grade retention, special education need, and school drop-out (4, 10, 11), which place them at risk for educational and occupational difficulties during adulthood. Indeed, a growing body of literature has documented a wide range of long-term consequences associated with ADHD such as low self-esteem, high-risk behaviors, disrupted relationships, delinquency, substance use, unemployment, lifelong disadvantage, and the development of other mental disorders (e.g., antisocial disorders, depression, anxiety, learning disabilities) (12–16). For instance, studies suggest that between 25 and 50% of children with ADHD have a comorbid learning disorder, with rates ranging from 18 to 45% for reading disabilities, 9 to 63% for writing difficulties, and 11 to 30% for math-related deficits (17–21). This comorbidity augments the risk for academic failure, since subjects with SLD have also been found to earn poorer GPA, require more educational support, and have lower high school and postsecondary completion rates (22, 23). In addition, poor readers are more vulnerable to mental health problems and negative outcomes than their peers, including low self-concept, anxiety, depression, attempted suicide, unemployment, and incarceration (22–26). Consequently, children exhibiting reading, math, or spelling difficulties in combination with attentional deficits could be much more impaired in learning than those with SLD or ADHD alone (27). In this vein, some authors found that subjects who meet criteria for both ADHD and SLD usually display more severe neurocognitive deficits in executive functions and working memory, negative academic experience, and higher risk of school retention than those with either isolated condition (18, 28, 29).

On the other hand, demographic characteristics such as gender, SES, stressful events (e.g., adoption, parental divorce/separation, grade retention), and lifestyle behaviors (e.g., diet, screen time, sleep duration) are also important variables that affect academic performance in children (30–35). In particular, school failure and poor academic achievement are strongly related to socioeconomic factors operating at individual and community levels (36–38). In this sense, multiple studies have reported the beneficial effects of living in a high-SES neighborhood on school readiness, verbal ability, reading skills, math achievement, and GPA, even after accounting for individual and family characteristics (36–38). Neighborhood affluence has also been positively associated with youth’s chances of completing high school, attending college, and years of schooling completed (36). By contrast, children and adolescents from low SES communities are at high risk for negative educational outcomes, including reading impairment, school drop-out, and academic underachievement (36, 38, 39). Of note, Fluss et al. (39) examined the prevalence of reading disabilities in 1,062 children distributed across three educational zones (i.e., low, medium, and high-SES) based on multiple social and demographic indicators, such as the parents’ professional background, the rate of unemployment in the district area, the percentage of disadvantaged families, and the proportion of non-native speakers living in the proximity of the school. The authors revealed that reading impairment was highly influenced by neighborhood SES, with estimates ranging from 3.3% in high SES to 24.2% in low SES areas (39). Besides, a strong association exists between living in disadvantaged communities and the occurrence of other psychiatric disturbances, including behavioral/emotional problems, ADHD, and depression (36, 40–42).

Thus, literature consistently shows that ADHD, SLD, and SES affect a host of educational outcomes in children and adolescents (4, 22, 36). However, there are still controversial issues that warrant further investigation. First, most studies featured relatively small samples, focused on academic attainment at the end of compulsory schooling, and relied on screening measures or self-reports for assessing the presence of ND instead of standardized diagnostic methods. Second, previous research on the relation between ADHD, SLD, and academic impairment has rarely examined these ND simultaneously. Likewise, additional confounding factors, such as gender, age, stressful events and lifestyle behaviors, have not been accounted for in the majority of investigations. Last, there have been no studies examining whether SES moderates the association between ADHD, SLD, and the academic achievement of youth on specific domains (e.g., first language, foreign language, mathematics). To address this gap in the literature and overcome some of the methodological limitations of earlier investigations, the current research aimed to evaluate for the first time the impact of ADHD and SLD on academic performance in 1,287 Spanish children distributed across a low-middle (LM)-income and a high-income population, when adjusted for comorbidity and demographic variables that may influence educational functioning (i.e., gender, age, nationality, adoption, parental divorce/separation, grade retention, dietary habits, screen use, sleep duration). The specific objectives were as follows: (a) to compare the prevalence of psychopathology, learning difficulties, and ND across a LM-income and a high-income population, (b) to examine whether SES, ADHD, and SLD were significantly related to academic performance, and (c) to determine whether the association between these ND and educational outcomes was modified by SES. We hypothesized that mental health problems, including psychiatric symptoms, learning difficulties, and ND would be more common among individuals from the LM-income group. We also hypothesized that lower SES would be associated with poor performance across multiple domains. Further, we expected that ADHD and SLD would increase the risk for academic impairment. Finally, we hypothesized that SES would moderate the relation between ADHD, SLD, and educational outcomes, such that the negative effect of ND on academic performance would be mitigated for children living in high SES areas. In the context of the extensive early and long-term consequences associated with such ND, it is important to determine potential factors that may buffer or worsen the academic difficulties faced by students with ADHD and SLD in order to design and implement targeted interventions that improve the academic achievement of those particularly vulnerable. Thus, results from this study will serve a practical use for clinicians, education researchers, and policymakers in their efforts to prevent school failure and promote students’ mental health by identifying those individuals who are at higher risk of underperforming.

## Materials and methods

### Participants

The current cross-sectional population-based sample is part of a larger research called INSchool, which started in 2011 with the aim of identifying children and adolescents’ mental health problems in a school setting. For the current investigation, we used data from six different schools, which participated in this ongoing research during the academic years 2015–2016 and 2016–2017.

The schools were equally distributed between two different territories of the Barcelona Metropolitan Area: Rubí, a LM-income area with 74,536 inhabitants, and Sant Cugat del Vallès, a high-income area with 87,830 inhabitants (43). Socioeconomic level was established based on a territorial socioeconomic indicator (TSI) that measures the socioeconomic characteristics of the Catalan population according to the employment status (i.e., percentage of employed residents, percentage of employed residents in blue-collar occupations), educational level (i.e., percentage of illiterate people or with primary education, percentage of residents with a junior school degree or below), immigration (i.e., percentage of foreign people from LM-income countries), and household income of the individuals who live in a small geographic unit (44). A reference value for Catalonia is 100 and a value for each territorial unit is established in comparison with the average Catalan value. In 2015, the mean TSI for the LM-income and high-income area was 97.8 and 124.7, respectively, which corresponded to the fourth and tenth decile. Data for both territorial units were obtained from the Statistical Institute of Catalonia (45).

The LM- and the high-income sample included 836 and 694 participants, respectively. Overall, 51.8% (*n* = 793) were boys and 48.2% were girls, with a mean age of 9.83 years (*SD* = 2.92; range = 5–17 years). Demographic characteristics for the total sample and by SES are summarized in Table 1.

**Table 1:** Demographic characteristics of the study samples.

	Total sample (*n* = 1,530)	LM-income sample (*n* = 836)	High-income sample (*n* = 694)	Statistic	Effect size	Value of *p*
Gender (*n*, %)				*χ*^2^ = 4.53	Cramer’s *V* = 0.054	0.033
Boys	793 (51.8)	454 (54.3)	339 (48.8)			
Girls	737 (48.2)	382 (45.7)	355 (51.2)			
Age (*M*, *SD*)	9.83 (2.92)	9.74 (2.89)	9.95 (2.95)	NS	NS	NS
Educational stage (*n*, %)				NS	NS	NS
Primary	918 (60.0)	517 (61.8)	401 (57.8)			
Secondary	612 (40.0)	319 (38.2)	293 (42.2)			
Nationality (*n*, %)				*χ*^2^ = 14.8	Cramer’s *V* = 0.098	< 0.001
Spanish	1,462 (95.8)	814 (97.6)	648 (93.6)			
Foreign origin	64 (4.19)	20 (2.40)	44 (6.36)			
Adoption (*n*, %)				*χ*^2^ = 13.7	Cramer’s *V* = 0.097	< 0.001
No	1,434 (98.8)	786 (99.7)	648 (97.6)			
Yes	18 (1.24)	2 (0.25)	16 (2.41)			
Parental divorce/separation (*n*, %)				*χ*^2^ = 7.41	Cramer’s *V* = 0.071	0.006
No	1,140 (78.0)	602 (75.3)	538 (81.3)			
Yes	321 (21.0)	197 (24.7)	124 (18.7)			
Frequency of screen use (*M*, SD)	2.11 (0.72)	2.12 (0.73)	2.10 (0.71)	NS	NS	NS
Sleeping hours (*M*, *SD*)	9.55 (0.98)	9.47 (0.99)	9.67 (0.95)	*t* = −3.66	Cohen’s *d* = 0.208	< 0.001
Three meals a day (*n*, %)				*χ*^2^ = 14.1	Cramer’s *V* = 0.096	< 0.001
No	134 (8.78)	94 (11.3)	40 (5.79)			
Yes	1,392 (91.2)	741 (88.7)	651 (94.2)			

Differences in sample sizes across variables are due to missing data. For *χ*^2^ tests with degrees of freedom equal to 1, the effect size is regarded as small for values > 0.10, moderate for values between 0.30 and 0.50, and strong for values > 0.50. With regard to Cohen’s *d*, a value of 0.20 is considered small, 0.50 medium, and 0.80 large. LM, low-middle; *M*, mean; NS, non-significant; *SD*, standard deviation.

### Procedure

**Figure 1:**
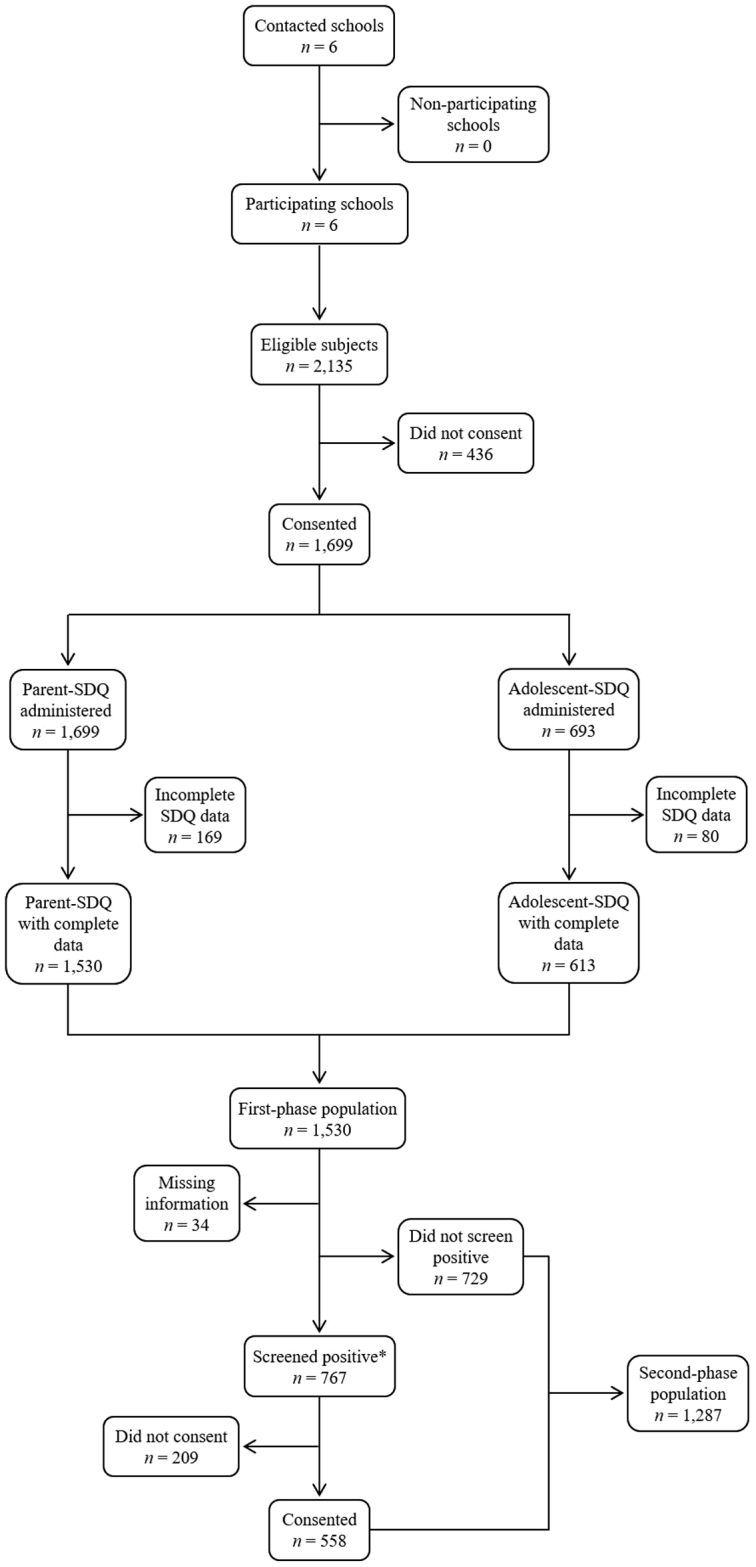
Flowchart of the study design and data collection process. *Cases were defined according to: a score in the clinical range on any of the parent-reported SDQ problem scales; five or more high-risk indicators on the PRODISCAT; or a previous diagnosis of neurodevelopmental disorder from a medical professional.

The Department of Health and the Department of Education (Generalitat de Catalunya, Spain) authorized the project, and ethical approval was granted by the Ethics Committee of the Vall d’Hebron Hospital Universitari (PR(AG)72-2012), in Barcelona. Six schools from the Barcelona Metropolitan Area were contacted in two academic years (i.e., 2015–2016, 2016–2017) and invited to participate after explaining the study to the school staff. All of them accepted, which included four public primary and two secondary schools equally distributed across a LM-income and a high-income area. All students enrolled in the selected schools, with ages comprised between 5 and 17 years, were considered for study inclusion, resulting in 2,135 eligible subjects. A two-stage procedure was applied: in the first phase of the project (i.e., from September to April), families were informed and we obtained informed consent for 1,699 children (participation rate = 79.6%), 693 of whom were at least 11 and also gave permission. Parents of the participating students received a questionnaire regarding demographic data and school-related factors along with the Strengths and Difficulties Questionnaire (SDQ) (46), which was completed at home. Additionally, the main classroom teachers provided information on reading and writing difficulties through the Protocol for Detection and Management of Dyslexia. Teacher’s Version (PRODISCAT) (47), and youth over 11 years completed the self-reported SDQ. Cases with missing values in the SDQ were removed. Thus, the final sample of this first phase comprised 1,530 children and adolescents. Of them, 767 were identified as potential cases in accordance with the following criteria: (a) a score in the clinical range on any of the parent-reported SDQ problem scales (i.e., Emotional symptoms, Conduct problems, Hyperactivity/inattention, Peer problems); (b) five or more high-risk indicators on the PRODISCAT; or (c) a previous diagnosis of ND from a medical professional as reported by parents. Subjects who screened positive were subsequently invited to participate in the second phase of the project (i.e., from January to June). Parents provided consent for 558 of them (participation rate = 72.8%) and children were interviewed for diagnostic confirmation based on the Diagnostic and Statistical Manual of Mental Disorders (DSM) criteria. Interviews were carried out on separate days within the school facilities so all students could complete the clinical assessment, which avoided sample attrition. Participants along with their parents met a psychiatrist of the research team, who determined the presence of ADHD using the Kiddie Schedule for Affective Disorders and Schizophrenia Present and Lifetime Version (K-SADS/PL) (48). Besides, students were granted an additional appointment with an experienced neuropsychologist who conducted a comprehensive neuropsychological battery for the evaluation of SLD, in addition to a full exploration of their cognitive abilities using the Wechsler Intelligence Scale for Children, fourth (WISC-IV) (49) or fifth edition (WISC-5) (50). The following standardized tests were administered to evaluate the reading and writing performance of youth: Battery for the Evaluation of Reading Processes, Revised (PROLEC-R) (51), Battery for the Evaluation of Reading Processes in Junior and Senior High-School Students, Revised (PROLEC-SE-R) (52), Test for the Analysis of Reading and Writing (TALE) (53), and Battery for the Evaluation of Writing Processes (PROESC) (54). In total, the second-phase population included students with a negative screening score and those who underwent the diagnostic assessment (*n* = 1,287). Finally, the information on academic performance was obtained from school records at the end of the academic year. Figure 1 describes the study design and data collection process.

### Measures

#### Demographic factors

Demographic data included child’s gender, age, country of birth, adoption, parental divorce/separation, and whether he or she had repeated any grade. Parents were asked if their child had ever received a diagnosis of ADHD, SLD, or another developmental disorder from a medical professional. They also provided information about their child’s eating habits by indicating how often they had breakfast, lunch and dinner (1 = *never*, 2 = *occasionally*, and 3 = *always*), and reported how frequently the student played video games, used the cell phone, and social networks. Each of these three categories had four possible responses (1 = *never*, 2 = *sometimes*, 3 = *often*, and 4 = *always*) and were summed to compute the mean frequency of screen use. Finally, sleep duration was derived from the answer to the following questions: “At what time does your child usually go to bed?” and “At what time does your child usually wake up?”

#### Strengths and difficulties questionnaire

The SDQ (46) is a screening instrument that can be administered to parents of children aged 4–17 or as self-reports in subjects over 11 years old. The questionnaire covers common areas of social, emotional, and behavioral functioning through 25 items distributed across five subscales: Emotional symptoms, Conduct problems, Hyperactivity/inattention, Peer problems, and Prosocial behavior. Each dimension consists of five items with three response options (0 = *not true*, 1 = *somewhat true*, and 2 = *certainly true*), thus yielding a score that ranges between 0 and 10. Furthermore, a Total difficulties score may be derived by summing the first four subscales (range = 0–40). The parent and adolescent versions of the SDQ used in the present study have been validated as feasible instruments to identify mental health problems in children aged 5–17 by Español-Martín et al. (55), who also provided Spanish normative data according to the child’s gender, age and type of informant. The frequency of subjects with a score in the clinical range on each SDQ subscale was estimated based on the bands developed by those authors (55). Ordinal alpha coefficients yielded adequate reliability estimates across all subscales, with values ranging from 0.76 to 0.89, both for the parent and adolescent version.

#### Protocol for detection and management of dyslexia: Teacher’s version

The PRODISCAT (47) is a screening instrument aimed at teachers and professors of primary and secondary education to detect children who present reading and/or writing difficulties. It is available in five different versions, depending on the educational stage, with good internal consistency (Cronbach’s alpha = 0.75–0.95) (56). These include 23–44 items, some of which are high-risk indicators that require intervention (e.g., “He/she has difficulties in lexical access when speaking,” “He/she makes many spelling mistakes compared to the class group,” “His/her reading speed is slow compared to the class group”). Each item has two response options (0 = *no*, 1 = *yes*). In the present study, subjects with five or more high-risk indicators on the PRODISCAT were regarded as potential cases of dyslexia.

#### Kiddie schedule for affective disorders and schizophrenia present and lifetime version

The K-SADS/PL (48) is a semi-structured interview that assesses current and past psychopathology in children and adolescents according to the DSM. It contains an introductory interview with questions about basic demographic characteristics, complaints, and prior psychiatric problems, followed by an 82-symptom screen interview and five diagnostic supplements: (a) Affective disorders, (b) Psychotic disorders, (c) Anxiety disorders, (d) ADHD and behavioral disorders; and (e) Substance abuse, tic, eating, and elimination disorders. Items are scored using a 0- to 3-point scale, where 0 means no information is available, 1 suggests the symptom is not present, 2 indicates subthreshold levels of symptomatology, and 3 represents threshold criteria. The diagnostic supplement for a given area is only completed if the child receives at least one threshold rating on any of the symptoms surveyed in that section of the screen interview. The Spanish version of the K-SADS/PL, which has shown good psychometric properties (57, 58), was administered to parents and children/adolescents separately.

#### Battery for the evaluation of reading processes, revised

The PROLEC-R (51) is one of the most extensively used instruments to assess reading performance in Spanish children aged 6–12 years. The battery explores the perceptual, lexical, syntactic, and semantic processes involved in reading comprehension through nine tasks, namely: Name or sound of letters, Equal-different, Words reading, Pseudo-words reading, Grammatical structures, Punctuation, Sentences comprehension, Text comprehension, and Listening comprehension. Adequate values of internal consistency, indexed by ordinal alpha, have been reported for all subtests (range = 0.76–0.95) (51). Reading accuracy and speed are measured by the number of correct answers and the time spent to complete each task.

#### Battery for the evaluation of reading processes in junior and senior high-school students, revised

The PROLEC-SE-R (52) evaluates the reading ability and the underlying lexical, syntactic, and semantic processes of adolescents from 12 to 18 years. The battery includes 13 tests (i.e., Lexical selection, Semantic categorization, Word reading, Pseudo-word reading, Grammatical structures I, Grammatical judgment, Grammatical structures II, Punctuation, Expository comprehension, Narrative comprehension, Pure reading comprehension, Mnemonic reading comprehension, Listening comprehension), with ordinal alpha coefficients ranging from 0.73 to 0.99 (52).

#### Test for the analysis of reading and writing

The TALE (53) allows to determine the general level and specific characteristics of reading and writing in children from first to fourth grade of primary school (6–10 years). It contains a battery of subtests, which evaluate the reading of letters, syllables, words and texts, as well as reading comprehension. The writing assessment consist of copying syllables, words and sentences, a dictation task, and writing a composition. In addition, it includes a part to examine aspects related to graphology, such as letters size and form, separation between words, etc. The number of mistakes and the time required to complete each task are compared to those from the general population.

#### Battery for the evaluation of writing processes

The PROESC (54) aims to evaluate the main writing processes in children from third grade of primary to adolescents in secondary education (8–15 years old). The battery is composed of six subtests (i.e., Dictation of syllables, Dictation of words, Dictation of pseudo-words, Dictation of sentences, Writing a story, Writing an essay) and has demonstrated good internal consistency for the total score (Cronbach’s alpha = 0.82) (54). The student’s writing performance is determined by comparing the number of correct answers in each subtest to the scale for the corresponding school year.

#### Academic performance

Academic performance on four core subjects (i.e., Catalan, Spanish, English as foreign language, and mathematics) was obtained from school records at the end of the academic year. However, schools varied in the way they scored those subjects and, thus, grades were converted into a 4-point scale from D to A (D = *unsatisfactory achievement*, *fail*, 0–4.9; C = *satisfactory achievement*, *pass*, *average*, 5–6.9; B = *good achievement*, *above average*, 7–8.9; and A = *excellent achievement*, 9–10). Given the high correlation between Catalan and Spanish grades (*r* = 0.75, *p* < 0.001), we calculated the academic performance on first language as the mean of the two scores.

### Statistical analyses

All analyses were performed with SPSS 22.0. Descriptive statistics were calculated to illustrate the demographic characteristics, clinically relevant cases based on the SDQ, academic performance, and prevalence of ADHD and SLD in the total sample and by SES. Individuals from the LM-income and the high-income population were compared with the *χ*^2^ test for categorical variables and Cramer’s *V* was used to estimate the strength of the significant associations. For continuous variables, we examined the normality and homoscedasticity of data using skewness, kurtosis, and Levene’s test. The skewness and kurtosis values were all within ±2, suggestive of normal distribution, and Levene’s test determined the homogeneity of variances (*p* > 0.05) (59, 60). Therefore, the Student’s *t*-test was applied for comparison of means, and Cohen’s *d* was reported as the effect size measure.

#### Data analytic plan

In order to determine whether there was evidence of clustering within the data, we first ran an intercept-only model for each educational outcome (i.e., grades on first language, foreign language, mathematics) with school as a random effect. Results from the unconditional models justified the use of a single-level approach, since the variance components for school were not significant, indicating that grades within schools were not different than grades between schools. A series of backward regressions were then performed to remove non-significant demographic predictors, followed by multiple ordinal logistic regressions that retained the significant demographic predictors identified in the preliminary analysis and added the primary variables of interest as described below. Analyses were conducted separately for each educational outcome, with A as the lowest category and D as the highest.

##### Preliminary analyses

Gender, age, nationality, adoption, parental divorce/separation, grade retention, frequency of screen use, sleep duration, and having three meals a day were entered into a series of backward regressions. Significant predictors in each model (*p* < 0.05) were retained.

##### Primary analyses

To examine the impact of ADHD and SLD on academic achievement, and the hypothesized moderating role of SES, a series of ordinal logistic regressions were performed. As noted above, the significant demographic predictors from the preliminary analyses were entered in Step 1. ADHD and SLD (Step 2), and SES (Step 3) were then added to test main effects, while controlling for comorbidity. Last, we included interaction terms (i.e., ADHD × SES, SLD × SES) into the final model (Step 4) and conducted stratified analyses in case of significant interactions. The Nagelkerke *R*^2^ was used to measure the global predictive capacity of each model. Odds ratio (*OR*) and their corresponding 95% confidence interval (*CI*) were reported. A two-sided *p*-value of 0.05 was set as significance level in all tests.

## Results

### Sample characteristics

The LM- and the high-income sample included 836 and 694 subjects, respectively. Overall, 51.8% (*n* = 793) of the participants were boys and 48.2% were girls, with a mean age of 9.83 years (*SD* = 2.92; range = 5–17 years). The proportion of boys was higher in the LM- than in the high-SES sample (54.3% vs. 48.8%, *p* = 0.033), while foreign origin was more prevalent among children from the high-income group (6.36% vs. 2.40%, *p* < 0.001). The proportion of participants reporting parental divorce/separation, adoption, and not having three meals a day was larger in the LM-income population (Table 1). Finally, no significant differences were detected in the frequency of screen use (Table 1), although subjects from the high-SES sample slept more hours than those from the LM-income group (9.67 vs. 9.47, *p* < 0.001).

### Prevalence of psychiatric symptoms and learning difficulties

Table 2 presents the proportion of children who fell within the clinical range on each SDQ subscale in the total sample and by SES. According to both parents’ and adolescents’ perception, LM-income students exhibited a higher prevalence of clinically relevant symptoms in all the SDQ subscales, with the exception of the Hyperactivity/inattention scale (Table 2). Besides, we identified a total of 349 (28.0%) participants who struggled in reading or writing based on the PRODISCAT. Of note, learning difficulties were significantly more frequent among individuals with lower SES (34.7% vs. 18.5%, *p* < 0.001).

**Table 2:** Clinically relevant cases according to the parent- and adolescent-reported SDQ.

	Total sample	LM-income sample	High-income sample	Statistic	Effect size	Value of *p*
SDQ parent (*n*, %)						
Emotional symptoms	244 (15.9)	163 (19.5)	81 (11.7)	*χ*^2^ = 17.3	Cramer’s *V* = 0.106	< 0.001
Conduct problems	269 (17.6)	174 (20.8)	95 (13.7)	*χ*^2^ = 13.3	Cramer’s *V* = 0.093	< 0.001
Hyperactivity/inattention	212 (13.9)	125 (15.0)	87 (12.5)	NS	NS	NS
Peer problems	234 (15.3)	143 (17.1)	91 (13.1)	*χ*^2^ = 4.67	Cramer’s *V* = 0.055	< 0.031
Total difficulties	176 (11.5)	120 (14.4)	56 (8.07)	*χ*^2^ = 14.7	Cramer’s *V* = 0.098	< 0.001
SDQ adolescents (*n*, %)						
Emotional symptoms	80 (14.3)	53 (18.5)	27 (9.93)	*χ*^2^ = 8.41	Cramer’s *V* = 0.123	0.004
Conduct problems	89 (15.9)	64 (22.4)	25 (9.19)	*χ*^2^ = 18.1	Cramer’s *V* = 0.180	< 0.001
Hyperactivity/inattention	71 (12.7)	38 (13.3)	33 (12.1)	NS	NS	NS
Peer problems	93 (16.7)	57 (19.9)	36 (13.2)	*χ*^2^ = 4.50	Cramer’s *V* = 0.090	0.034
Total difficulties	63 (11.3)	41 (14.3)	22 (8.09)	*χ*^2^ = 5.43	Cramer’s *V* = 0.099	0.020

For *χ*^2^ tests with degrees of freedom equal to 1, the effect size is regarded as small for values > 0.10, moderate for values between 0.30 and 0.50, and strong for values > 0.50. LM, low-middle; NS, non-significant; SDQ, Strengths and Difficulties Questionnaire.

### Academic performance

As shown in Table 3 there were significant differences on academic performance according to SES, although repeater students were equally distributed across the LM- and the high-income population (5.28% vs. 4.01%, *p* = 0.249). In particular, students who achieved a grade of A or B were over-represented within the high-income sample for all educational outcomes (i.e., first language, foreign language, mathematics). By contrast, children from the LM-income population received lower grades, except on foreign language where the number of students with a grade of D was greater in the high-income group.

**Table 3:** Academic performance on first language, foreign language, and mathematics.

	Total sample (*n* = 1,530)	LM-income sample (*n* = 836)	High-income sample (*n* = 694)	Statistic	Effect size	Value of *p*
Grade retention (*n*, %)				NS	NS	NS
No	1,419 (95.3)	772 (94.7)	647 (96.0)			
Yes	70 (4.70)	43 (5.28)	27 (4.01)			
First language (*n*, %)				*χ*^2^ = 27.6	Cramer’s *V* = 0.135	< 0.001
A	216 (14.3)	102 (12.3)	114 (16.6)			
B	516 (34.1)	249 (30.0)	267 (39.0)			
C	732 (48.3)	444 (53.6)	288 (42.0)			
D	50 (3.30)	34 (4.10)	16 (2.34)			
Foreign language (*n*, %)				*χ*^2^ = 32.0	Cramer’s *V* = 0.146	< 0.001
A	227 (15.0)	107 (13.0)	120 (17.5)			
B	502 (33.2)	243 (29.5)	259 (37.8)			
C	691 (45.8)	432 (52.4)	259 (37.8)			
D	90 (5.96)	43 (5.21)	47 (6.86)			
Mathematics (*n*, %)				*χ*^2^ = 40.1	Cramer’s *V* = 0.163	< 0.001
A	227 (15.0)	87 (10.5)	140 (20.4)			
B	464 (30.7)	238 (28.8)	226 (33.0)			
C	708 (46.9)	432 (52.4)	276 (40.3)			
D	111 (7.35)	68 (8.24)	43 (6.28)			

Differences in sample sizes across variables are due to missing data. For *χ*^2^ tests with degrees of freedom equal to 2, the effect size is regarded as small for values > 0.07, moderate for values between 0.21 and 0.35, and strong for values > 0.35. LM, low-middle; NS, non-significant.

### Prevalence rates of ADHD and SLD

According to parent reports, the prevalence of students who already had a diagnosis of ND prior to the start of the study was 9.25% (*n* = 119) and did not differ by SES (LM-income: 10.3%; high-income: 7.88%, *p* = 0.131). Based on the diagnostic assessment conducted in the second phase, however, a total of 282 (21.9%) subjects met criteria for at least ADHD or SLD and significant differences were found between the LM- and the high-income sample (25.6% vs. 17.3%, *p* < 0.001). Specifically, 123 students were identified as having ADHD, which represented an overall prevalence rate of 9.56%. Of these, 63 (4.90%) met criteria for the combined presentation, 4.20% (*n* = 54) had the predominantly inattentive presentation, and 0.47% (*n* = 6) were diagnosed with the predominantly hyperactive–impulsive presentation. The prevalence of SLD was 16.4% (*n* = 211), with reading and writing difficulties being present in 15.4% (*n* = 198) and 6.29% (*n* = 81) of the total sample, respectively. Notably, children from the LM-income population appeared to have a significantly higher prevalence of SLD (20.7% vs. 11.0%, *p* < 0.001) and reading difficulties (19.7% vs. 10.0%, *p* < 0.001), while the rate of writing difficulties (LM-income: 7.40%; high-income: 4.90%, *p* = 0.067) and ADHD (LM-income: 8.66%; high-income: 10.7%, *p* = 0.220) was similar across samples. Last, over 18% (*n* = 52) of the students who received a diagnosis of ND suffered from both ADHD and SLD. Thus, the overall prevalence was 4.04% and did not differ by SES (LM-income: 3.77%; high-income: 4.38%, *p* = 0.582).

### Demographic predictors of academic performance

As a preliminary analysis, we removed non-significant demographic predictors of academic performance following the backward elimination process for each educational outcome (i.e., grades on first language, foreign language, mathematics). Results indicated that grades on first language and foreign language were negatively associated with male gender (*p* < 0.001), parental divorce/separation (*p* = 0.005 and *p* < 0.001, respectively), grade retention (*p* < 0.001), frequency of screen use (*p* = 0.008 and *p* = 0.038, respectively), and not having three meals a day (*p* < 0.001 and *p* = 0.012, respectively); age, nationality, and adoption, on the other hand, were not retained (*p* > 0.05) for the primary analyses described below. Significant predictors of poor academic performance on mathematics included age (*p* < 0.001), parental divorce/separation (*p* < 0.001), grade retention (*p* < 0.001), and not having three meals a day (*p* = 0.038), while gender, nationality, adoption, frequency of screen use, and sleep duration were not retained (*p* > 0.05) (Tables 4–6; Step 1).

**Table 4:** Predictors of poor academic performance on first language.

	Nagelkerke *R*^2^	*OR* (95% *CI*)	Value of *p*
Step 1	0.146		
Gender (ref. Girls)		1.75 (1.37–2.25)	< 0.001
Parental divorce/separation (ref. No)		1.57 (1.14–2.14)	0.005
Grade retention (ref. No)		13.6 (6.52–28.3)	< 0.001
Frequency of screen use		1.27 (1.07–1.52)	0.008
Three meals a day (ref. Yes)		2.46 (1.54–3.94)	< 0.001
Step 2	0.277		
Gender (ref. Girls)		1.77 (1.40–2.25)	< 0.001
Parental divorce/separation (ref. No)		1.47 (1.09–1.98)	0.011
Grade retention (ref. No)		10.3 (4.98–21.1)	< 0.001
Frequency of screen use		1.28 (1.08–1.52)	0.004
Three meals a day (ref. Yes)		2.34 (1.49–3.66)	< 0.001
ADHD (ref. No)		3.94 (2.48–6.26)	< 0.001
SLD (ref. No)		6.39 (4.33–9.45)	< 0.001
Step 3	0.283		
Gender (ref. Girls)		1.74 (1.37–2.20)	< 0.001
Parental divorce/separation (ref. No)		1.46 (1.09–1.96)	0.012
Grade retention (ref. No)		10.4 (5.07–21.3)	< 0.001
Frequency of screen use		1.28 (1.08–1.52)	0.004
Three meals a day (ref. Yes)		2.18 (1.39–3.41)	0.001
ADHD (ref. No)		4.14 (2.60–6.59)	< 0.001
SLD (ref. No)		6.07 (4.10–8.96)	< 0.001
SES (ref. High-income sample)		1.41 (1.12–1.78)	0.003
Step 4	0.283		
Gender (ref. Girls)		1.73 (1.36–2.19)	< 0.001
Parental divorce/separation (ref. No)		1.44 (1.07–1.94)	0.016
Grade retention (ref. No)		10.4 (5.00–21.4)	< 0.001
Frequency of screen use		1.28 (1.08–1.52)	0.004
Three meals a day (ref. Yes)		2.20 (1.40–3.45)	0.001
ADHD (ref. No)		3.83 (2.02–7.29)	< 0.001
SLD (ref. No)		6.89 (4.26–11.1)	< 0.001
SES (ref. High-income sample)		1.38 (1.08–1.78)	0.011
ADHD × SES		NS	NS
SLD × SES		NS	NS

All steps are incremental. First, the significant demographic predictors were entered in Step 1. ADHD and SLD (Step 2), and SES (Step 3) were then added to test main effects, while controlling for comorbidity. Last, we included interaction terms (i.e., ADHD × SES, SLD × SES) into the final model (Step 4) to examine the potential moderating role of SES. ADHD, attention-deficit/hyperactivity disorder; *CI*, confidence interval; NS, non-significant; *OR*, odds ratio; SES, socioeconomic status; SLD, specific learning disorders.

**Table 5:** Predictors of poor academic performance on foreign language.

	Nagelkerke *R*^2^	*OR* (95% *CI*)	Value of *p*
Step 1	0.156		
Gender (ref. Girls)		1.72 (1.37–2.16)	< 0.001
Parental divorce/separation (ref. No)		1.70 (1.29–2.26)	< 0.001
Grade retention (ref. No)		17.2 (9.37–31.8)	< 0.001
Frequency of screen use		1.18 (1.01–1.39)	0.038
Three meals a day (ref. Yes)		1.72 (1.13–2.63)	0.012
Step 2	0.293		
Gender (ref. Girls)		1.73 (1.37–2.19)	< 0.001
Parental divorce/separation (ref. No)		1.61 (1.21–2.16)	0.001
Grade retention (ref. No)		15.5 (7.94–30.3)	< 0.001
Frequency of screen use		1.20 (1.01–1.41)	0.033
Three meals a day (ref. Yes)		1.58 (1.02–2.46)	0.042
ADHD (ref. No)		3.50 (2.27–5.40)	< 0.001
SLD (ref. No)		6.68 (4.60–9.70)	< 0.001
Step 3	0.296		
Gender (ref. Girls)		1.70 (1.33–2.17)	< 0.001
Parental divorce/separation (ref. No)		1.60 (1.18–2.17)	0.002
Grade retention (ref. No)		15.5 (7.74–31.1)	< 0.001
Frequency of screen use		1.19 (1.00–1.42)	0.045
Three meals a day (ref. Yes)		NS	NS
ADHD (ref. No)		3.60 (2.29–5.67)	< 0.001
SLD (ref. No)		6.43 (4.35–9.51)	< 0.001
SES (ref. High-income sample)		NS	NS
Step 4	0.299		
Gender (ref. Girls)		1.71 (1.34–2.18)	< 0.001
Parental divorce/separation (ref. No)		1.60 (1.18–2.17)	0.002
Grade retention (ref. No)		14.9 (7.41–30.0)	< 0.001
Frequency of screen use		1.19 (1.01–1.42)	0.044
Three meals a day (ref. Yes)		NS	NS
ADHD (ref. No)		2.58 (1.40–4.73)	0.002
SLD (ref. No)		5.56 (3.54–8.75)	< 0.001
SES (ref. High-income sample)		1.36 (1.05–1.77)	0.020
ADHD × SES		NS	NS
SLD × SES		NS	NS

All steps are incremental. First, the significant demographic predictors were entered in Step 1. ADHD and SLD (Step 2), and SES (Step 3) were then added to test main effects, while controlling for comorbidity. Last, we included interaction terms (i.e., ADHD × SES, SLD × SES) into the final model (Step 4) to examine the potential moderating role of SES. ADHD, attention-deficit/hyperactivity disorder; CI, confidence interval; NS, non-significant; OR, odds ratio; SES, socioeconomic status; SLD, specific learning disorders.

**Table 6:** Predictors of poor academic performance on mathematics.

	Nagelkerke *R*^2^	*OR* (95% *CI*)	Value of *p*
Step 1	0.130		
Age		1.12 (1.08–1.16)	< 0.001
Parental divorce/separation (ref. No)		1.88 (1.43–2.47)	< 0.001
Grade retention (ref. No)		7.35 (4.18–12.9)	< 0.001
Three meals a day (ref. Yes)		1.51 (1.02–2.23)	0.038
Step 2	0.211		
Age		1.11 (1.07–1.15)	< 0.001
Parental divorce/separation (ref. No)		1.80 (1.40–2.32)	< 0.001
Grade retention (ref. No)		5.96 (3.48–10.2)	< 0.001
Three meals a day (ref. Yes)		NS	NS
ADHD (ref. No)		2.71 (1.88–3.92)	< 0.001
SLD (ref. No)		3.47 (2.58–4.68)	< 0.001
Step 3	0.231		
Age		1.11 (1.07–1.16)	< 0.001
Parental divorce/separation (ref. No)		1.78 (1.37–2.31)	< 0.001
Grade retention (ref. No)		5.99 (3.45–10.4)	< 0.001
Three meals a day (ref. Yes)		NS	NS
ADHD (ref. No)		2.97 (2.03–4.33)	< 0.001
SLD (ref. No)		3.20 (2.35–4.35)	< 0.001
SES (ref. High-income sample)		1.80 (1.46–2.22)	< 0.001
Step 4	0.231		
Age		1.11 (1.07–1.16)	< 0.001
Parental divorce/separation (ref. No)		1.79 (1.37–2.32)	< 0.001
Grade retention (ref. No)		5.96 (3.42–10.4)	< 0.001
Three meals a day (ref. Yes)		NS	NS
ADHD (ref. No)		2.91 (1.72–4.91)	< 0.001
SLD (ref. No)		3.02 (2.09–4.36)	< 0.001
SES (ref. High-income sample)		1.85 (1.47–2.31)	< 0.001
ADHD × SES		NS	NS
SLD × SES		NS	NS

All steps are incremental. First, the significant demographic predictors were entered in Step 1. ADHD and SLD (Step 2), and SES (Step 3) were then added to test main effects, while controlling for comorbidity. Last, we included interaction terms (i.e., ADHD × SES, SLD × SES) into the final model (Step 4) to examine the potential moderating role of SES. ADHD, attention-deficit/hyperactivity disorder; *CI*, confidence interval; NS, non-significant; *OR*, odds ratio; SES, socioeconomic status; SLD, specific learning disorders.

### ADHD, SLD, and SES as predictors of academic performance

#### First language

After controlling for gender, parental divorce/separation, grade retention, frequency of screen use, and daily meals (Table 4; Step 1), ADHD and SLD were significant risk factors for academic underachievement (Table 4; Step 2). Specifically, children who received a diagnosis of ADHD had ~4 times the odds of achieving lower grades on first language (*OR* = 3.94, 95% *CI* = 2.48–6.26, *p* < 0.001), while those with SLD were over 6 times more likely to perform poorly (*OR* = 6.39, 95% *CI* = 4.33–9.45, *p* < 0.001). SES was also found to predict academic achievement (Table 4; Step 3), suggesting that LM-income students performed significantly worse than those from the high-income population (*OR* = 1.41, 95% *CI* = 1.12–1.78, *p* = 0.003). These findings remained in Step 4 (ADHD: *OR* = 3.83, 95% *CI* = 2.02–7.29, *p* < 0.001; SLD: *OR* = 6.89, 95% *CI* = 4.26–11.1, *p* < 0.001; SES: *OR* = 1.38, 95% *CI* = 1.08–1.78, *p* = 0.011), where we added interaction terms into the final model to examine the potential moderating role of SES. However, there were no significant interactions between ADHD or SLD and SES (Table 4), indicating that these ND exert a pervasive impact on educational outcomes across different socioeconomic backgrounds.

#### Foreign language

After controlling for gender, parental divorce/separation, grade retention, frequency of screen use, and daily meals (Table 5; Step 1), ADHD and SLD increased the risk for academic impairment (Table 5; Step 2), while SES failed to predict underachievement (Table 5; Step 3). However, with the interaction terms included in the model (Step 4), subjects from the LM-income population were found to achieve lower grades on foreign language than those with higher SES (*OR* = 1.36, 95% *CI* = 1.05–1.77, *p* = 0.020). Furthermore, the effects of ADHD and SLD were still significant in Step 4 (ADHD: *OR* = 2.58, 95% *CI* = 1.40–4.73, *p* = 0.002; SLD: *OR* = 5.56, 95% *CI* = 3.54–8.75, *p* < 0.001) and did not differ by SES, since no significant interactions were observed (Table 5).

#### Mathematics

After controlling for age, parental divorce/separation, grade retention, and daily meals (Table 6; Step 1), ADHD and SLD were negatively related to academic achievement on mathematics (Table 6; Step 2). Step 3 also revealed a significant main effect of SES, since LM-income students tended to perform worse than those from the high-income population (*OR* = 1.80, 95% *CI* = 1.46–2.22, *p* < 0.001). These associations remained mainly the same when interaction terms were introduced in Step 4 (ADHD: *OR* = 2.91, 95% *CI* = 1.72–4.91, *p* < 0.001; SLD: *OR* = 3.02, 95% *CI* = 2.09–4.36, *p* < 0.001; SES: *OR* = 1.85, 95% *CI* = 1.47–2.31, *p* < 0.001), although the interaction between ADHD or SLD and SES was not significant (Table 6). Thus, higher SES did not attenuate the risk for academic underachievement conferred by these ND.

## Discussion

The current research examined whether ADHD and SLD were significantly related to academic achievement across specific subjects (i.e., first language, foreign language, mathematics) in a sample of 1,287 Spanish children from a LM- and a high-income population, and whether the association was modified by SES, when controlling for comorbidity and demographic factors that may influence educational functioning.

In accordance with previous studies (36, 39–42, 61), we observed a higher prevalence of emotional/behavioral problems, learning difficulties, and ND among children from the LM-income population, which supported the hypothesis that low SES would be associated with negative health outcomes. Interestingly, a prior investigation conducted in a representative sample of Catalan children aged 4–14 also showed that those from disadvantaged families were at risk of worse mental health than their counterparts, as evidenced by higher scores on most of the SDQ subscales (41). On the other hand, these findings replicate the increased likelihood of learning disabilities found in low SES communities (23, 40, 62, 63), since the rate of SLD and reading difficulties was approximately two-fold higher within the LM-income students (20.7% and 19.7%, respectively). Likewise, Fluss et al. (39) reported that reading impairment was highly influenced by neighborhood SES, with estimates ranging from 3.3% in high SES to 24.2% in low SES areas, and poor children have shown to be 1.5 times more likely to have a learning disability (39, 64). In this sense, it has been hypothesized that children from more disadvantaged communities might have less access to learning materials, cultural resources and stimulating environments, which would limit their reading skills and literacy experiences, particularly if parents lack the tools to supplement their education (36, 37, 39, 63). Besides, the current research adds to a wealth of data documenting the positive relation between SES and academic achievement (36–38). As we expected, students within the high-income sample had better grades across all educational outcomes, and lower SES increased the likelihood of poor academic performance.

Our findings also supported the hypothesis that ADHD would increase the risk for academic impairment and are consistent with previous literature, showing that youth with ADHD experience a variety of academic difficulties, including poor grades, low academic achievement, special education need, grade retention and failure to complete high school, even after adjusting for intelligence, SES, and learning disabilities (10, 11, 65). Interestingly, though, the present study confirms for the first time the association between ADHD diagnosis and academic performance in Spain and supports a prior analysis conducted by Pagerols et al. (33), which found that students with increased levels of attention problems were more likely to perform poorly, regardless of other risk factors such as comorbid psychopathology, sociodemographic characteristics, stressful events, and lifestyle behaviors (33). Moreover, the current investigation showed that SLD contributed independently to heighten the risk of academic underachievement, especially for language subjects, suggesting that learning disorders may compromise the academic performance of students by impeding or slowing their acquisition of knowledge through reading (22). Thus, children with diagnosed learning disabilities have been found to earn lower GPA, require more educational support, and have higher dropout rates than their peers (22, 23, 66).

Overall, these results confirmed that ADHD, SLD and, to a lesser extent, low SES are significant predictors of poor performance across multiple academic domains, even after adjustment for comorbidity and demographic variables that may influence educational functioning (i.e., gender, age, parental divorce/separation, grade retention, frequency of screen use, dietary habits). However, studies that examine the role of SES on academic achievement among children who suffer from these ND are surprisingly sparse. Indeed, the current research was the first to investigate whether high SES might attenuate the association between ADHD, SLD and adverse educational outcomes. Given the lack of significant interactions with SES and the strongest main effect observed for ND in all the academic domains assessed, our findings indicate that SES does not provide the hypothesized buffer against the negative impact of ADHD and SLD on academic performance. Similarly, a recent study, which evaluated whether the association between a clinical diagnosis of depression and lower educational attainment was modified by SES, did not find significant moderating effects (67).

Considering that ADHD and SLD, unlike SES, are more easily modifiable risk factors, these results have large public health implications as the ADHD- and SLD-related academic problems can lead to both short- and long-term consequences, including lower productivity, unemployment, economic hardship, stress, anxiety, depression, and low self-esteem (5, 68–70). Thus, early identification and effective intervention strategies aimed at children with these ND might improve their educational functioning and mitigate later negative outcomes. In this sense, there is evidence for a positive short-term effect of ADHD medications on some aspects of school performance (e.g., improvements in classroom behaviors, seatwork productivity, GPA and achievement testing), although long-term studies are scarce and effects are generally smaller or more diverse (4, 71). This suggests that additional supports both in the family and academic setting may be necessary to effectively remediate the academic difficulties faced by students with ADHD and SLD. Particularly, teachers may play an important role in the educational development of affected children as evidenced by studies showing that school-based interventions enhance the academic achievement of children and adolescents with ADHD mainly by optimizing their classroom behavior, self-regulation, organizational skills, and homework performance (72, 73).

The current research should be interpreted in the light of some strengths and limitations. One of the main strengths is that, for the first time to our knowledge, we investigated the impact of ADHD and SLD on children’s academic performance across specific domains (i.e., first language, foreign language, mathematics) in Spain, and whether SES moderates the association between ND and educational outcomes after adjusting for comorbidity and demographic variables that may influence educational functioning. Additional advantages include the large size and age range of the sample, composed of 1,287 students aged 5–17 years, and the comprehensive case identification through the administration of standardized screening instruments and DSM-based clinical interviews by trained psychiatrics and neuropsychologists. Moreover, our study involved the use of real-life measures of academic performance in a school-based sample, which allowed the detection of undiagnosed children and provides a more valid reflection of the general population than a clinical sample. Finally, we controlled for multiple demographic risk factors and limited shared method variance by using different informants for clinical diagnoses and academic achievement (74). Nevertheless, alternative explanations for the observed relations cannot be excluded since other possible confounding variables, such as children’s intelligence quotient, executive functioning, treatment status or other psychiatric comorbidities, were not considered. Similarly, the cross-sectional design of the study prevents from drawing conclusions on causality. Students who screened negative did not undergo the clinical assessment and, therefore, false negatives might have occurred. In addition, we cannot discount the possibility of a selection bias with regard to subjects included and excluded from the analysis. Last, the SES measure used in our study may have misrepresented the individual SES of participants and resulted in different patterns of associations (75, 76). Yet, area-level indicators of SES provide useful information on contextual factors that are relevant to health beyond individual or family-level characteristics (75, 76). For instance, there is evidence that neighborhood conditions may affect a variety of outcomes, including educational attainment and employment, through the availability and quality of local services (e.g., child care centers, preschools, public schools, afterschool programs, health care facilities), physical and social environment stressors (e.g., air pollution, exposure to heavy metals, crime and violence levels, noise), and neighborhood-based networks (36, 77).

## Conclusions and future directions

Overall, the present investigation adds to the evidence for mental health disparities across socioeconomic backgrounds, and expands previous research on the relation between ND and academic performance by analyzing children from LM-income and high-income populations in order to determine whether SES moderates the association. Our results confirmed the higher prevalence of psychiatric symptoms, learning difficulties, and ND observed in disadvantaged communities. We also found that ADHD, SLD and, to a lesser extent, low SES are significant predictors of poor performance across multiple academic domains, even after adjustment for comorbidity and demographic variables that may influence educational functioning (i.e., gender, age, parental divorce/separation, grade retention, frequency of screen use, dietary habits). However, SES did not modify the association, suggesting that students with a clinical diagnosis of ADHD or SLD are more likely to achieve lower grades on first language, foreign language, and mathematics regardless of their socioeconomic background. Given the extensive consequences associated with academic problems, these findings highlight the need for early detection and intervention strategies aimed at students who suffer from ND in order to improve their functioning at school. In this sense, integrating mental health services within the school setting or in routine pediatric revisions could be helpful, especially for children from low SES communities who might have limited access to health care. Moreover, the identification of factors that may attenuate the risk for academic underachievement conferred by ADHD and SLD is imperative to design and implement targeted interventions for particularly vulnerable individuals. Therefore, future research should explore other potential moderators, such as parental involvement, personal attributes and social networks, educational support, or treatment type and length. Alternatively, studies examining whether the relation between ND and academic performance is modified by SES should include participants from more financially disadvantaged backgrounds, and combine individual measures of SES with data at the community level to simultaneously estimate individual and contextual effects. Further, a broader selection of covariates (e.g., intelligence, executive functioning, psychiatric comorbidities, treatment status) is required to fully validate the results of the current investigation.

## Data availability statement

The raw data supporting the conclusions of this article will be made available by the authors, without undue reservation.

## Ethics statement

The studies involving human participants were reviewed and approved by the Ethics Committee of the Vall d’Hebron Hospital Universitari, the Department of Health, and the Department of Education (Generalitat de Catalunya, Spain). Written informed consent to participate in this study was provided by the participants’ legal guardian/next of kin.

## Author contributions

GE-M, MP, and RB: study conception and design. GE-M, RP, and CR: data collection. GE-M, MP, and RP: data curation. GE-M and MP: data analysis and interpretation of results. JR-Q, MC, and RB: study supervision. MC: funding acquisition. GE-M and MP: writing–draft manuscript preparation. JR-Q, MC, and RB: writing–review and editing. All authors contributed to the article and approved the submitted version.

## Funding

This work was funded by Fundació “la Caixa,” Diputació de Barcelona, and Departament de Salut (Generalitat de Catalunya). The funders were not involved in the study design, collection, analysis, interpretation of data, the writing of this article or the decision to submit it for publication.

## Conflict of interest

GE-M has received travel grants from Angelini Pharma, Laboratorios Rubió, Lundbeck, and Takeda for participating in psychiatric meetings. JR-Q has served on the speakers’ bureau and acted as consultant for Bial, Bristol Myers Squibb, Janssen-Cilag, Laboratorios Raffo, Laboratorios Rubió, Medice, Novartis, Shionogi, Shire, Sincrolab, Takeda, Tecnofarma, and Uriach. He has received travel awards from Bial, Janssen-Cilag, Laboratorios Rubió, Medice, Shionogi, Shire, and Takeda for participating in psychiatric meetings. The Department of Psychiatry chaired by JR-Q has received unrestricted educational and research support from Janssen-Cilag, Laboratorios Rubió, Oryzon, Psious, Roche, and Shire in the past 3 years. MC has received travel grants and research support from Eli Lilly and Co., Janssen-Cilag, and Shire. He has been on the advisory board and served as a consultant for Eli Lilly and Co., Janssen-Cilag, and Shire.

The remaining authors declare that the research was conducted in the absence of any commercial or financial relationships that could be construed as a potential conflict of interest.

## Publisher’s note

All claims expressed in this article are solely those of the authors and do not necessarily represent those of their affiliated organizations, or those of the publisher, the editors and the reviewers. Any product that may be evaluated in this article, or claim that may be made by its manufacturer, is not guaranteed or endorsed by the publisher.
